# Diagnosing homo digitalis: towards a standardized assessment for digital tool competencies

**DOI:** 10.3389/fpsyg.2023.1270437

**Published:** 2024-01-04

**Authors:** Sarah E. M. Stoll, Isabel Bauer, Karen Hopfer, Judith Lamberty, Verena Lunz, Francesca Guzmán Bausch, Cosima Höflacher, Gregory Kroliczak, Solène Kalénine, Jennifer Randerath

**Affiliations:** ^1^Department of Psychology, University of Konstanz, Konstanz, Germany; ^2^Lurija Institute for Rehabilitation Science and Health Research, Kliniken Schmieder, Allensbach, Germany; ^3^Department of Developmental and Educational Psychology, Faculty of Psychology, University of Vienna, Vienna, Austria; ^4^Cognitive Neuroscience Center, Action and Cognition Laboratory, Faculty of Psychology and Cognitive Science, Adam Mickiewicz University, Poznan, Poland; ^5^Department of Clinical Neuropsychology, Nicolaus Copernicus University in Toruń Collegium Medicum, Bydgoszcz, Poland; ^6^Sciences Cognitives Et Sciences Affectives, University of Lille, Lille, France; ^7^Outpatient Unit for Research, Teaching, and Practice, Faculty of Psychology, University of Vienna, Vienna, Austria

**Keywords:** digital tools, aging, digital competencies, assessment, neurorehabilitation, inclusion, digital literacy, novel tools

## Abstract

**Introduction:**

In the 21st century, digital devices have become integral to our daily lives. Still, practical assessments designed to evaluate an individual’s digital tool competencies are absent. The present study introduces the “Digital Tools Test” (“DIGI”), specifically designed for the evaluation of one’s proficiency in handling common applications and functions of smartphones and tablets. The DIGI assessment has been primarily tailored for prospective use among older adults and neurological patients with the latter frequently suffering from so-called apraxia, which potentially also affects the handling of digital tools. Similar to traditional tool use tests that assess tool-selection and tool-action processes, the DIGI assessment evaluates an individual’s ability to select an appropriate application for a given task (e.g., creating a new contact), their capacity to navigate within the chosen application and their competence in executing precise and accurate movements, such as swiping.

**Methods:**

We tested the implementation of the DIGI in a group of 16 healthy adults aged 18 to 28 years and 16 healthy adults aged 60 to 74 years. All participants were able to withstand the assessment and reported good acceptance.

**Results:**

The results revealed a significant performance disparity, with older adults displaying notably lower proficiency in the DIGI. The DIGI performance of older adults exhibited a correlation with their ability to employ a set of novel mechanical tools, but not with their ability to handle a set of familiar common tools. There was no such correlation for the younger group.

**Conclusion:**

In conclusion, this study introduces an innovative assessment tool aimed at evaluating common digital tool competencies. Our preliminary results demonstrate good acceptance and reveal expected group differences. For current cohorts of older adults, the results seem to indicate that the ability to use novel tools may aid digital tool use. In the next step, the psychometric properties of the DIGI assessment should be evaluated in larger and more diverse samples. The advancement of digital tool competency assessments and rehabilitation strategies is essential when we aim at facilitating societal inclusion and participation for individuals in affected populations.

## Introduction

In daily life and society, Information Communication Technologies like the internet, smartphones, tablets, and applications (apps) have become ubiquitous. Proficiency in these technologies and broad digital competencies are important assets for participation in the working world (Oberländer et al., 2020). The concept of “digital competence” was recognized as one of the eight core competencies for lifelong learning by the European Parliament and Council as early as 2006 (European Parliament, 2006). Its significance extends beyond the professional world since the activities of daily living are increasingly shaped by digitalization. In numerous aspects of our lives, digital technologies have emerged as the most convenient means of access. For example, in the realm of transportation and travel, we simply call an Uber via an app or find the nearest subway station with the “maps” application on our smartphones. These digital approaches offer advantages, including flexibility and mobility (Quamar et al., 2020). Arguably, one of the most pivotal roles played by modern digital technologies is in the domain of communication. Instant messaging (e.g., WhatsApp, Messenger), email services, social networking platforms (e.g., Instagram, Facebook) and video conferencing (e.g., Skype, Zoom) nowadays are common (Quamar et al., 2020). Typically, these communication tools are accessed through the use of smartphones.

The role of participation in digital opportunities is particularly evident across different demographic groups. Among the younger population, aged between 20 and 25, digital tools have emerged as the primary medium for communication. In fact, owning a smartphone is considered by this age group as an almost indispensable component of social interaction, and those without such a device are perceived to be partially excluded from these interactions (Möller, 2016). A longitudinal study in a Finnish sample showed that also in middle-aged and older persons, the perceived necessity to own and use information and communication technology (such as smartphones and tablets) was growing (Wilska and Kuoppamäki, 2017). In the case of older adults and individuals with medical conditions, especially in the context of eHealth and mHealth (i.e., the provision of healthcare services through Information Communication Technologies, particularly smart mobile devices), the significance of these technologies has been steadily growing. As preventive measures or complements to traditional medical care, mobile health apps are becoming increasingly accessible via smartphones or tablets. Unfortunately, the adoption of smart mobile devices is still less prevalent in older age groups, even though older adults may benefit the most from telemedical apps and mHealth communication (Chiarini et al., 2013; Li et al., 2014; Changizi and Kaveh, 2017). To close these gaps there is a need to analyze potential factors contributing to non-use.

One important factor for non-use or inappropriate use presents an inadequate understanding of how to properly operate these devices. There are several potential challenges older adults might face when attempting to navigate digital devices. First, there appear good news when looking at overall usability of smartphones and tablets. Kortum and Sorber (2015) who investigated usability ratings of the most popular applications on iOS and Android OS among more than 3,000 participants reported high usability ratings. Also in the older population, there is a positive reception of smart mobile technologies: in a recent study, Brunzini et al. (2023) found that older Italian citizens regarded digital devices, including smartphones and tablets, as quite useable and learnable. Moreover, their small pilot sample demonstrated only few errors when operating these devices for social support, and entertainment purposes. However, usability and performance measures frequently seem to dissociate in older adults. For example, in a study comparing touchscreen versus keyboard use in two tasks, Sonderegger et al. (2016) found that while older adults were equally effective at solving text input- and menu selection-tasks as their younger counterparts, they performed less efficient. At the same time the perceived usability of smartphones was rather positive in older adults. Multiple obstacles faced by senior citizens were identified by McGaughey et al. (2013) or Gomez-Hernandez et al. (2023) in their reviews: Some difficulties can be attributed to the device itself, such as the small size of the gadget, others depend on characteristics of the user, such as physical and cognitive limitations or a lack of confidence and training. Furthermore, studies suggest that age, together with educational background, may have an influence on the ability to solve technology-associated problems (Ertl et al., 2020).

However, non-use due to reduced competencies does not merely pertain to healthy older adults, but also to persons with cognitive disabilities, for example after stroke. We propose that digital tool competencies is also a highly relevant topic in the context of neurorehabilitation. Strikingly, limb apraxia, known as a disorder of (traditional) tool use (Goldenberg, 2013; Randerath, 2023), is a frequent consequence of brain damage such as stroke with a prevalence of 28–37% among stroke survivors (Donkervoort et al., 2000). The term “limb apraxia” refers to disorders of learned and purposeful movements (Liepmann, 1900; Heilman and Rothi, 1993). When applying the traditional tool use assessments using the DILA-S in stroke patients (Buchmann and Randerath, 2017; Buchmann et al., 2020a,b), our patients’ left us with the impression that next to their common tool competencies (i.e., how to use a fork or a toothbrush), their digital tool competencies (i.e., send a note or picture to their relatives using a messenger-application) are just as important to them for their ADLs (activities of daily living) and participation. From our observations, there are valid concerns surrounding the capacity of stroke patients to navigate digital devices. Lastly, apraxia is only one of a vast variety of potential syndromes and disorders after stroke that may affect digital tool use. Other stroke-associated symptoms affecting motor, perceptual, communicative, or cognitive abilities such as hemiplegia, hemineglect, aphasia, and deficits concerning concentration and memory are potential influencing factors that also may detrimentally impact digital competencies. Another concern regarding the capacity of stroke patients to operate digital devices relates to the advanced age of many individuals in this patient group (Busch and Kuhnert, 2017). Therefore, it is important to first investigate the digital tool use competencies in healthy older adults.

Considering the profound impact of Information Communication Technologies on ADLs, in their review Quamar et al. (2020) conclude that it “marks a paradigm shift in the way we assess and measure everyday functioning”. The digitalization drives the need for standardized tests of basic digital skills to be considered for ADL assessments, contributing to the “paradigm shift”.

The assessment of individual difficulties in common digital tool competencies seems an important step towards characterizing an individual’s problem also before offering a tailored training intervention. There are strong efforts to enhance digital accessibility for the older population, e.g., by designing special user interfaces for older adults (Arab et al., 2013; Sakdulyatham et al., 2017) or providing smartphone training classes (Zhao et al., 2020). A standardized assessment could be useful to evaluate the success of such an intervention. Despite the decent amount of tests for general or specific technological knowledge and skills among high school and college students (for an overview see Covello and Lei, 2010), standardized instruments for the assessment of digital competencies in the general population are scarce. Existing assessments rely on self-report questionnaires rather than practical tasks (Ferrari, 2013; Lu et al., 2017; Karnoe et al., 2018; Zhao et al., 2020) or they focus on device usability (Sonderegger et al., 2016; Brunzini et al., 2023).

Inspired by our clinical work, we developed a novel pragmatic assessment for digital tool competencies. The major goal of this manuscript is to introduce the so-called DIGI (DIGItal tools test). This instrument aims to assess fundamental digital tool competencies focusing on elementary tasks associated with the utilization of smart mobile devices, namely smartphones and tablets. It evaluates participants’ performance regarding their ability to select an adequate application (selection), successful navigation inside the application (production) and minimize motor-related errors (motor error). The DIGI has been developed especially for use in older adults and neurologic patients. In the present pilot study, we sought to test the feasibility and acceptance of the DIGI in a sample of healthy young and older adults participating in the assessment. The study further involved a comparative analysis between the two cohorts and included a correlational analysis with performance in the traditional novel and familiar tool use tests of the Diagnostic Instrument for Limb Apraxia (DILA-S).

### The current study

Despite unprecedented opportunities of smart mobile devices in supporting independence and healthcare for older adults and neurological patients, many older individuals are hesitant to use these devices due to a lack of competence or because brain damage may have impaired their ability to use these tools. A prerequisite for administering adequate digital tool use training is the standardized assessment of abilities and difficulties in handling smart mobile devices. Currently, there is a lack of a suitable assessment tool for this purpose. In the current study, we aim to address this gap by introducing a newly developed assessment for evaluating digital tool use competencies, named DIGI. This assessment evaluates a set of everyday skills and tasks in operating smartphones or tablets, like saving a contact or connecting the device to the power socket for charging. Performance is evaluated based on correctly choosing (selection) and using (production) the essential features to handle each task, as well as on movement-related mistakes (motor error).

We anticipated no drop outs, good acceptability and that the group of older adults will show significantly more difficulties in handling digital devices compared to the younger group with significantly lower selection and production scores and significantly more movement-related mistakes than the younger group. We further explored whether the proficiency to use traditional novel versus familiar tools would correlate with the ability to use modern smart mobile devices in the young as well as in the older group.

## Methods

The study was approved by the ethics committee of the University of Konstanz (#15/2020) and conducted in accordance with the declaration of Helsinki. All participants gave informed consent before taking part in the study. Post hoc power-analyses can be found in the Supplementary material, Table 1.

### Participants

Data was collected from March 2019 to July 2019. The younger sample consisted of 16 subjects ranging between 18 and 28 years (*M* = 23.50, *SD* = 2.68), with half of them being female. The older adults sample included 16 participants, aged between 60 and 74 years (*M* = 64.25, *SD* = 3.99), with nine of them being female. None of the participants showed signs of cognitive impairment as evidenced by their DemTect Scores ≥13 (Kalbe et al., 2004). Two subjects, one in each group, indicated to be left-handed. Hand sensibility, assessed with the two-point discrimination test (for a detailed description see Hunter et al., 1990) did not differ between the older and younger group (*U* = 119.5, *z* = −0.34, *p* = 0.752).

### DIGI

For a comprehensive description of the DIGI, please refer to the manual, booklets and evaluation sheets (available at https://kops.uni-konstanz.de/entities/publication/09b43e22-1e78-4561-9833-9eaa7963f38f). The DIGI was developed to assess the skills in handling digital devices. During the assessment, participants are tasked with completing everyday-like assignments using a smart mobile device. The experimenter evaluates the participant’s performance using an evaluation sheet, considering the successful selection of an adequate application, the effective navigation inside the application and the skillfulness of the motor movement when interacting with the device. The DIGI assessment consists of two versions, denoted as A and B, which cater to both, smartphone, and tablet, compatible with the operating systems iOS and Android. Booklets and evaluation sheets are available for both operating systems. In the present pilot study, all participants used the Android-based devices. Each of the two versions comprises the same two practice trials (see AB 00.1 and AB 00.2 in Table 1), eight tasks for smartphone and seven tasks for tablet. Parallel-items that were chosen for their close resemblance were: A01-B01; A02-B02; A03-B03; A04-B04; A05-B07; A06-B08; A07-B05; A08-B06 (please note, whether both subsets are solved in a similar manner will be looked at in a subsequent study evaluating psychometric properties by use of a larger sample). Notably, two tasks involving the phone function (A 02 *answer a call*, B 02 *make a call*) are exclusive to the smartphone version. The remaining tasks are identical for smartphone and tablet. Practice trials are excluded from the evaluation, since the experimenter may provide assistance to participants in completing them. Successful connection to the Wi-Fi and having saved a contact are prerequisites performing subsequent tasks. DIGI-tasks encompass various everyday skills and operations on smart mobile devices. A comprehensive list of all items can be found in Table 1.

**Table 1:** Overview of the tasks of the DIGI by versions A and B.

DIGI-A	Item	DIGI-B	Item
AB 00.1	Save contact	AB 00.1	Save contact
AB 00.2	Connect to Wi-Fi	AB 00.2	Connect to Wi-Fi
A 01	Charge the device	B 01	Connect the headphones
A 02	Answer a call	B 02	Make a call
A 03	Set an alarm	B 03	Save appointments
A 04	Send smiley	B 04	Send photo
A 05	Mute	B 05	Navigate
A 06	Take a photo	B 06	Zoom in
A 07	Open website	B 07	Set to flight mode
A 08	Zoom out	B 08	Delete photo

### Evaluation

**Figure 1:**
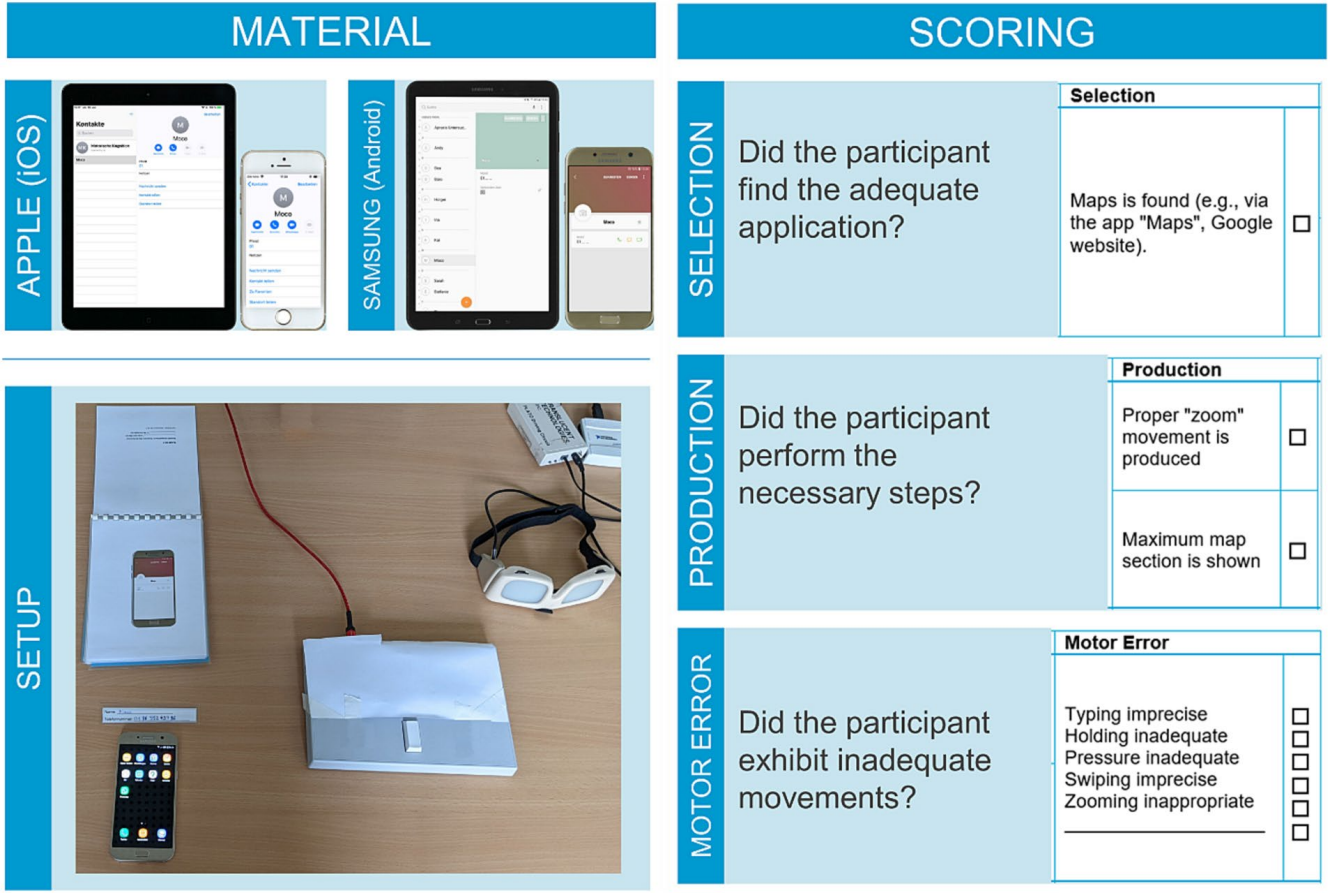
Setup and material of the DIGI. The top two left panels show the used devices (smartphones, tablets) and below the DIGI setup is displayed as used in the current study, including response pad and shutter goggles. On the right exemplary excerpts from the evaluation sheets including selection (top), production (middle) and error scores (bottom) are displayed. We would like to point out that the response pad, shutter goggles and head mounted camera are not necessary equipment to conduct the DIGI. At the current developmental stage of the assessment instrument, it served to control the timing of the visual input and the placement of the participant’s hand, as well as to refer to video footage of the test sessions whenever necessary (e.g., to test for interrater reliability). For the application in clinical practice, the DIGI requires only the smart mobile devices, booklets, and evaluation sheets.

An example of an evaluation sheet is displayed in Figure 1. Each item of the DIGI is evaluated based on two major criteria: the selection criterion and production criteria, which correspond to the process of app-selection and of navigating inside the application. The selection criterion pertains to the correct choice of the application suitable for the task (e.g., item *save contact*: input mask is reached (e.g., via contacts, telephone)). The production criteria evaluate the correct solution for each item in two action steps (e.g., for the item *save contact*: 1. Data input, 2. Save). Participants could achieve a maximum of 8 points (tablet: 7) per subtest for selection and 16 points (tablet: 14) for production. The separate evaluation of selection and production criteria is based on the finding that for traditional tools the selection and application can be impaired selectively in stroke patients (Buchmann and Randerath, 2017). Additionally, observable movement-related errors are documented. Typical observable movement errors include imprecise typing, inadequate holding, inadequate pressure, imprecise swiping, and inappropriate zooming. Notably, it is possible to record further movement-related errors. It also needs to be noted that future studies in this field should include kinematics-related evaluation procedures that allow for more precise movement tracking or objective movement error recognition. For each different observed error, one error-point is recorded. For example, if a participant’s typing and swiping are both imprecise in one trial, two error-points are noted.

### Material

The material and devices listed below and displayed in Figure 1 were employed for the implementation of the DIGI. The DIGI encompasses form sheets for evaluation, booklets displaying the current task with accompanying photographs showing the target end-state of the device, and paper flashcards with additional information necessary to solve the current task. Further materials include: a multi-socket, device-specific chargers, headphones, an object to be photographed (in this study a toy cat was utilized), and an iOS or Android smartphone and tablet. The smartphones and tablets used in the current study were a Samsung Galaxy A7 smartphone and a Samsung Galaxy Tab A tablet. In our laboratory, the DIGI is also available with an iPhone SE, and an iPad Air. Each device was equipped with a current Android OS or iOS version and received regular updates to ensure optimal functionality.

The primary objective of this study is to introduce the new assessment instrument, DIGI. Consequently, we will focus on the selection, production and motor-error scores. For experimental purposes a Cedrus Response Pad RB-540 was used in the present study. By instructing the participants to press a button of the Response Pad between trials with their hand which operated the digital device, the starting position of the hand was controlled. PLATO Visual Occlusion Spectacles from Translucent Technologies Inc. served to control the timing of visual input. These devices were controlled by a 15.6-inch laptop (ASUS VivoBook) running a Windows 10 Home operating system and the Cedrus Superlab 5 experimental software. To facilitate the evaluation of the participant’s performance, a head-mounted camera (GoPro Hero Session) was used to record screen activity during the DIGI.

### Procedure

In the course of this study, participants undertook the DIGI assessment using the Android operating system. Each participant completed the test on both, smartphone and tablet.

First, the participant put on the GoPro camera and the goggles. Then two practice trials were conducted followed by the DIGI tasks from versions A and B. The order in which versions and smart mobile devices were presented was balanced evenly among subjects. The response pad was placed adjacent to the hand operating the device. The digital device was placed centrally in front of the participant on the table, showing the home screen. The booklet was placed vertically to the device (see Figure 1). Between the trials, the goggles were shut and the participants placed their hand on the response pad’s key.

Each trial started with a verbal instruction of the respective task, consisting of a brief description (“Save contact”) and a specification of the task (“Save the number ‘…’ with the name ‘…’ in the contacts”). Participants were given the time they needed, i.e., the task was not time-constrained. Additionally, the booklet with a picture of the successful end-state of the device was presented for reference. This end-state is one of several possible solutions since some items can be solved in various acceptable ways. For example, in devices with Android OS, enabling flight mode may be accomplished via the settings menu – as shown in the booklet. However, it is also possible to enable flight mode via the taskbar, which usually can be dragged down from the upper edge of the home screen. This method results in a visually different, but correct end-state which is credited.

In the present study, the participants were allowed to use their preferred hand or both hands to solve the tasks.

### DILA-S

Subtests from the Diagnostic Instrument for Limb Apraxia were administered in this study (DILA-S, for material and manual).^1^ The results from the novel (NTT) and familiar (FTT) tools subtests are reported. In both NTT and FTT, participants first select the most appropriate tool from a set of three options and subsequently manipulate an object with the correct tool. The object is either a cylinder (NTT) that shall be lifted from a socket or a well-known everyday object (FTT) that shall be manipulated (e.g., scooping soup from a pot). Participants receive 0–2 selection points per trial, resulting in a total selection score for novel or familiar tool use between 0 and 10 points. Additionally, the participants’ ability to correctly manipulate the object is awarded with 0–2 execution-points per trial. This means that the range for the execution-score in NTT and FTT is 0–10 points. For a more comprehensive description of the DILA-S please see Buchmann and Randerath (2017).

### Acceptance

The acceptance of the DIGI has been assessed by use of an adapted version of the Akzept! questionnaire by Kersting (2008).

### Interrater reliability

Video recordings (received via GoPro) from a subsample of the older group (*n* = 7) were analyzed by a second independent rater who evaluated the participants’ performance in the DIGI. Selection, production, and motor-related error scores were summed up across DIGI versions A and B, smartphone, and tablet, and correlated between the experimenter and the independent rater using Kendall’s tau.

### Data analysis

The normality of the data was assessed on a group-wise basis by using the Kolmogorov–Smirnov test (K-S test). Results of the K-S test indicated that several variables from the DIGI, NTT, and FTT were not normally distributed in either age group (*p* < 0.05). Consequently, the non-parametric Mann–Whitney test was applied for between-group comparisons of DIGI selection-, production-, and error-scores. The Bonferroni-Holm procedure was applied to correct for multiple testing.

Correlations between the DIGI selection scores and the selection scores of the NTT and FTT were computed using Kendall’s tau. The same procedure was applied for the correlation of DIGI production scores and NTT and FTT execution scores. The correlations were conducted separately for each age group and for each device used.

## Results

### Analysis of group differences

**Figure 2:**
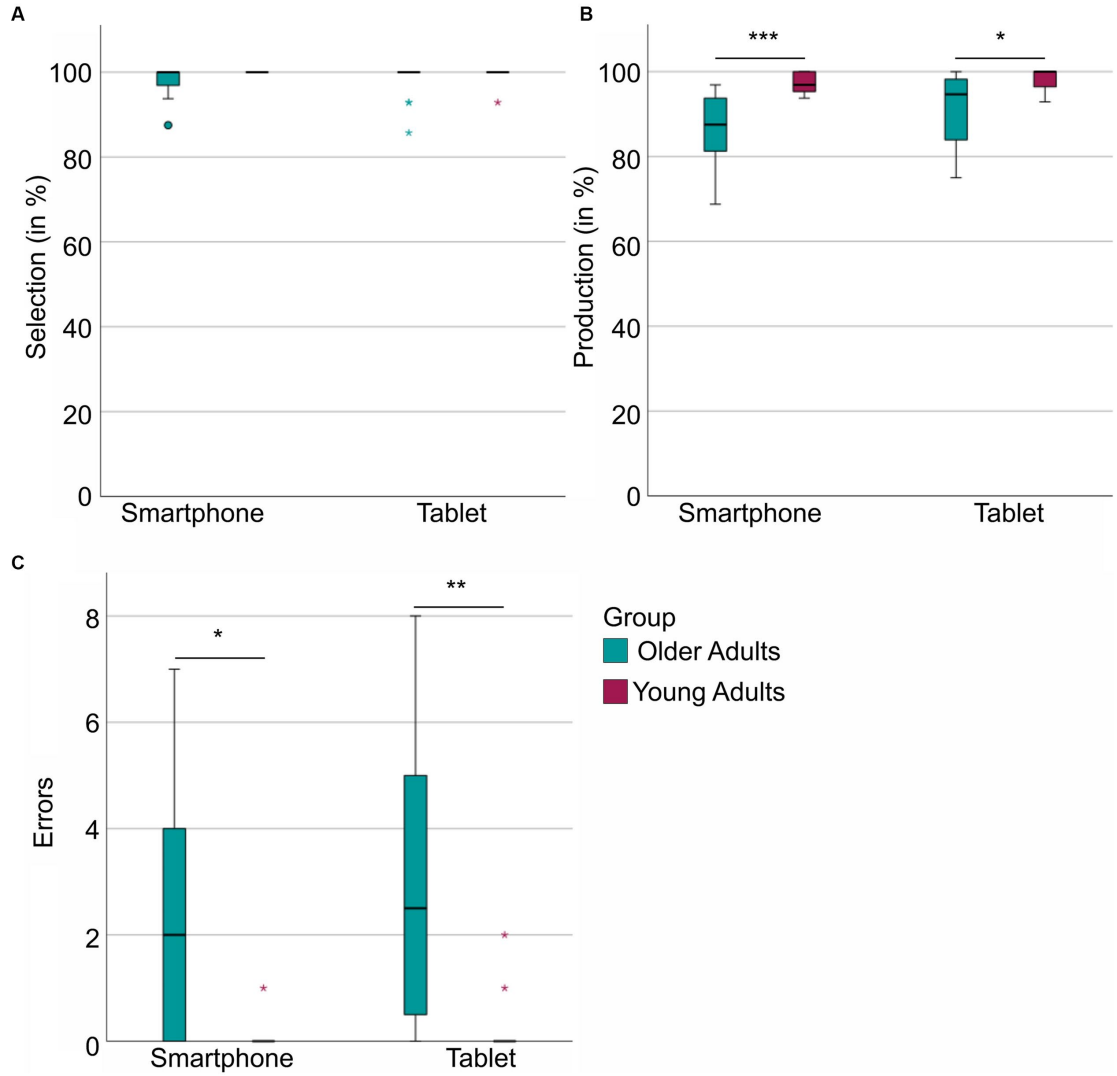
DIGI scores per group (older adults vs. young) and per device (smartphone vs. tablet). **(A)** Displays the DIGI selection score in percent per group and device. **(B)** The DIGI production score per group and device. **(C)** The sum of movement-related errors per group and device. ^*^p_adj_ ≤ 0.05, ^**^p_adj_ ≤ 0.01, ^***^p_adj_ ≤ 0.001 (adjusted with Bonferroni-Holm procedure).

For an overview of group comparisons concerning the DIGI variables, please see Table 2. Consistent with our hypothesis, we observed that the older age group achieved significantly lower production scores when operating the smartphone or the tablet. Furthermore, the older adults committed significantly more movement-related errors than the young adults on both devices. However, the selection score did not differ significantly between the age groups, for neither smartphone nor tablet (Figure 2).

**Table 2:** Comparisons between age groups on DIGI variables by use of the Mann–Whitney test.

Variable	Older (m; SD)	Young (m; SD)	*U*	*z*	*p*	*p* adj.
Smartphone selection score (in %)	98.05;0.3.76	100.00;0	160.00	2.10	0.239	0.478
Smartphone production score (in %)	86.91;7.67	97.46;2.61	241.50	4.35	<0.001	<0.001^***^
Smartphone error score	2.50;2.66	0.06;0.25	51.00	−3.43	0.003	0.012^*^
Tablet selection score (in %)	98.21;4.12	99.55;1.79	144.50	1.08	0.539	0.539
Tablet production score (in %)	91.10;8.71	98.66;2.21	201.00	2.93	0.005	0.015^*^
Tablet error score	3.13;2.85	0.19;0.54	40.00	−3.66	0.001	0.005^**^
Hand sensibility	2.56;0.79	2.38;0.39	119.50	−0.34	0.752	–

^*^p_adj_ ≤ 0.05, ^**^p_adj_ ≤ 0.01, ^***^p_adj_ ≤ 0.001 (adjusted with Bonferroni-Holm procedure).

### Correlation of digital and traditional tool use performance (Kendall’s tau)

In the younger age group, no significant correlation was identified between the DIGI scores and the performance in the NTT (selection *M* = 7.44; production *M* = 19.69; execution *M* = 9.13) and FTT (selection *M* = 9.53; production *M* = 19.60; execution *M* = 9.73), for both smartphone and tablet (*τ* ≤ 0.372, *p* ≥ 0.142). Correlations with the smartphone selection score could not be calculated due to a lack of variance in the younger age group.

**Figure 3:**
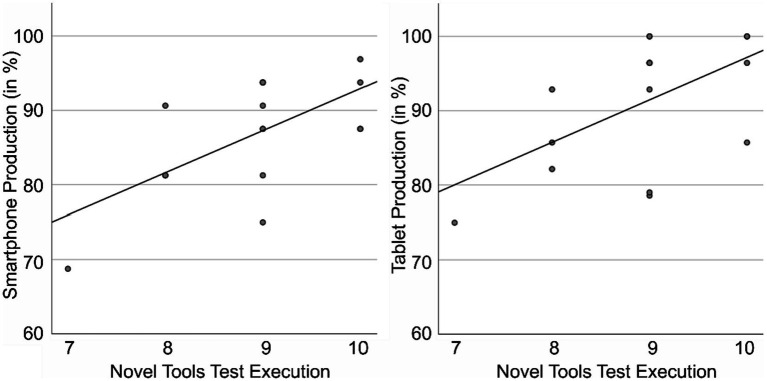
Correlation of smartphone and tablet production scores with NTT execution in the older age group.

Conversely, in the older age group, we observed a significant positive correlation between smartphone production score and NTT execution (execution *M* = 8.94) (*τ* = 0.44, *p* = 0.040), as well as between tablet production score and NTT execution (*τ* = 0.47, *p* = 0.028) (Figure 3). Except for these two, there were no further significant correlations between the FTT (selection *M* = 10.00; production *M* = 19.75; execution *M* = 9.75)/NTT (selection *M* = 7.75; production *M* = 19.81) and any of the DIGI variables (*τ* ≤ 0.081, *p* ≥ 0.728). Due to a lack of variance, no correlations could be calculated between FTT selection and the DIGI scores in the older age group.

### Acceptance

Participants rated the DIGI immediately for acceptance after completing the test items. Mean values and standard deviations for the single items of the acceptability questionnaire are shown in Table 3. There is an overall good acceptance of the DIGI as indicated by both groups. The DIGI has been graded by the older adults with 2.06 (SD = 0.75) and by the younger adults with 1.56 (SD = 0.54) according to the German grading system (1 indicates ‘very good’ and 6 indicates ‘insufficient’).

**Table 3:** Acceptance ratings for the DIGI per group.

	Older adults means (SD)	Young adults means (SD)
*Scale: 1 (does not apply) – 6 (applies completely)*		
The test tasks were clear and comprehensible.	5.88 (0.34)	5.50 (1.32)
The test can precisely map the differences that exist in relation to the tested characteristic.	5.13 (1.09)	5.13 (1.02)
The test tasks reflect the use of digital devices, which is also required in everyday life.	5.56 (0.89)	4.81 (1.60)
I felt overburdened during the test.	1.56 (1.09)	1.44 (1.26)
It is doubtful that the test will reveal difficulties in the use of digital devices.	2.25 (1.39)	2.31 (1.30)
The test reliably measures what it measures.	5.06 (1.18)	4.50 (1.32)
I did not understand the task.	1.00 (0.00)	1.31 (1.25)
Working through the test tasks is stressful.	1.69 (1.25)	1.38 (1.26)
I always knew what I had to do when working on the test tasks.	5.13 (1.45)	5.00 (1.86)
The ability to perform well in the tested tasks and the ability to use digital devices are two entirely different things.	2.50 (1.51)	2.25 (1.00)
The test allows you to precisely measure the differences in performance between different people in the ability covered by the test.^a^	4.80 (1.08)	4.63 (1.15)
The majority of the test tasks were too difficult for me.	1.19 (0.54)	1.06 (0.25)
The test tasks have too little in common with reality to accurately predict success in the use of digital devices.	1.44 (0.89)	1.69 (0.79)
Working through the test tasks is exhausting.	2.19 (1.80)	1.13 (0.34)
I did not understand the test tasks.	1.06 (0.25)	1.06 (0.25)
The test evaluation can provide an accurate picture of a person’s abilities.	4.69 (1.74)	4.81 (0.98)
*Scale: 1 (very good) – 6 (insufficient)*		
What grade would you give the test you just finished?	2.06 (0.93)	1.56 (0.51)
Compared to other people in my age group (with the same level of education), I think I did … in the test.	2.81 (0.75)	2.19 (0.54)

Items adapted from Akzept! by Kersting (2008) and translated from German. ^a^One missing value in the older group for this item (i.e., *n* = 15).

### Interrater reliability

We observed significant correlations between the experimenter’s and the independent rater’s evaluation of the participants’ performance in the DIGI on all three scores: Selection score (*τ* = 1.00, *p* < 0.001), production score (*τ* = 0.781, *p* = 0.015) and movement-related error scores (*τ* = 0.900, *p* = 0.006).

## Discussion

In the present work, we introduced the DIGI, an assessment tool for evaluating common competencies in handling smartphones and tablets. Through a pilot test involving a small sample of young and older adults, we demonstrated good interrater reliability, feasibility and acceptability of the DIGI assessment. We further showed its potential to detect performance differences in digital tool competencies between younger and older adults.

Our finding suggests that older adults might understand as proficiently as younger adults which application suits best for the assigned task. The older group was able to find and tap the appropriate app-icon on the mobile device and there were no differences between age groups in terms of selection scores.

However, consistent with our hypothesis, the older adults exhibited significantly more problems in producing the correct steps while navigating within the apps. This became evident in group differences for the DIGI production- and movement-related error scores for both smartphone and tablet. Common movement-related errors included, for example, imprecise typing whenever entering text and misperceptions about the meaning of a digital gesture, such as confusing typing and swiping when answering a phone call or confusing the zoom-in and the zoom-out gesture. It seems unlikely that this deficit could be explained by a decreased hand sensibility in the older age group since we observed that hand sensibility did not differ between groups. The literature, however, demonstrates that older adults show indeed a variety of motor deficits in comparison to younger adults, such as difficulties in coordination, increased variability of movements, slowing of movements, and difficulties with balance and gait, which are attributable to age-related changes in the central nervous system (for an overview see Seidler et al., 2010). These age-related changes in the central nervous system might have contributed to the increase in motor-related errors in the older age group. Early technology-related findings by Smith et al. (1999) support this hypothesis. The authors showed that cursor control tasks with a computer mouse were significantly more difficult for older than younger adults. In their study, this difficulty was associated with age-related declines in motor control, specifically in motor coordination. Comparable mechanisms might have led to the observation of more movement-related errors in the older group of the current study.

Furthermore, in the older age group, the ability to use digital tools correlated with the ability to use traditional (mechanical) but novel tools. Specifically, individuals with lower skills in navigating digital tools tend to display lower skills in applying novel tools to their recipient objects. This could point towards three different interpretations. One hypothesis posits that lower digital tool competencies are indicative of cognitive decline due to healthy aging. Substantiating this hypothesis, the existing literature demonstrates that digital app usage including such characteristics as number of apps used, usage by hour of day, swipes, and keystroke events predicts cognitive ability in older adults as measured with neuropsychological assessments (Gordon et al., 2019). The second hypothesis could point towards healthy older people having overall difficulties in novel hand-tool interactions in the sense of mechanical reasoning and thereby showing lower practical digital tool competencies. While previous results (Randerath et al., 2017) suggest that healthy older versus young subjects do not differ on a group level in performing novel tool use, the current study demonstrates a correlation between novel tool use and digital tool use skills. The third hypothesis directs towards an effect of the cohort with reduced familiarity with digital rules. Older people, who did not grow up surrounded by digital technologies, are sometimes labeled as “digital immigrants” (Prensky, 2001). They may need similar resources to handle digital tools as they need for using traditional (mechanical) novel tools. This may relate to general rule retrieval that is also discussed to be essential for novel tool utilization (Randerath, 2020; Stoll et al., 2022). The Broca area may be a relevant neural correlate that has been associated with different behavioral tasks based on rules, such as rule-guided actions (Bunge, 2004; Donohue et al., 2008), and grammatical rules in language syntax (Tettamanti et al., 2002). An overlap of lesion areas associated with impaired novel tool selection in Broca’s area have been discussed to be related to the retrieval and maintenance of object characteristics and physic rules (Stoll et al., 2022). The speculated potential overlap of digital tool competencies and behavioral and neural correlates of rule retrieval and novel tool use needs to be addressed in future studies. The argument that digital immigrants who encountered digital technology much later in life may approach these devices like novel tools is in line with the third hypothesis. Instead, younger people, commonly referred to as digital natives, may use different resources for digital competencies, relying more on common knowledge and overlearned procedures. In accordance with the hypothesis that the brains of digital natives might diverge from those of digital immigrants (Prensky, 2001), we speculate that the younger and the older age groups in our study might have recruited different areas of the brain to solve the DIGI. Participants in the older age group might have employed similar brain regions to solve the DIGI as they do to solve the DILA-S NTT. Our data implies that subsequent studies on the DIGI’s psychometric properties need to clarify its underlying constructs cohort-wise as age and the year born may both play a decisive role.

Furthermore, in our study, all participants utilized laboratory-owned smartphones and tablets rather than their personal devices. While this has several practical reasons (standardization, data protection, assessment procedure etc.) there are also some challenges going along with this. For example, a participant might have been familiar with the Android OS in general but running on a Huawei smartphone, and therefore, may not have been versed in its operation on a Samsung device, specifically on a Samsung Galaxy A7 used in the current setup. Dealing with unfamiliar devices can lead to user errors, given variations in the design and operation of different smartphones and tablets (Byrom and Row, 2017; Germine et al., 2019) and perhaps younger participants are more flexible in switching between brands.

It appears notable that the here-described difficulties in handling digital tools in the older sample may further extend to potential non-use of more specific health apps. The question arises of how to secure the inclusion and participation of those suffering from a loss of digital competencies. Digital tool use has gained growing importance not only for the area of health improvement but also in medical diagnostics. For example, current literature discusses approaches that target cognitive digital phenotyping by capturing everyday cognition *in vivo* via digital tool use (Hackett and Giovannetti, 2022). As some studies suggest that app use can predict cognitive performance decline (Gordon et al., 2019), the idea of cognitive digital phenotyping would be, for example, to contribute to early diagnosis of dementia by evaluating a person’s app using behavior (Hackett and Giovannetti, 2022). The inevitable growth in these approaches promises increasing gains and advantages but faces many challenges including participation of vulnerable groups.

There are certain methodological limitations and challenges when assessing digital tool competencies such as the handling of smartphones and tablets. It is important to keep the experimental devices in an up-to-date state to ensure their optimal functionality. However, this practice can introduce concerns regarding the comparability of early and later DIGI surveys, since the software, and the UI might change slightly with updates. Similarly, hardware, software, and the way we use it changes rapidly, which may pose a difficulty in the context of the thorough development of a neuropsychological diagnostic instrument (Schmand, 2019). Thus, it is questionable how long the specific tasks included in the DIGI will be relevant for our everyday living. Additionally, it is debatable for how long the specific smartphone−/tablet-brands we included in the DIGI will remain among the most frequently used ones. While we here provide a framework for presenting items and evaluating practical digital competencies of common tasks and features of smartphones and tablets, for future developments, we expect that regular reevaluations and adjustments of the items and devices present necessary steps.

Specific limitations of the current study are the small sample sizes and ceiling effects in certain DIGI and DILA-S variables especially in the young adults group. A major objective for future research is to enlarge our samples for all age groups and incorporate conditions with constraint hand use to obtain control samples for neurologic patients who oftentimes suffer from motor unimanual impairments such as hemiparesis. For our neurologic sample, it will be important to broaden the sample and include more severely impaired patients. The next steps entail collecting psychometric data and evaluating behavioral and neural correlates of diminished digital competencies.

## Conclusion

In light of the growing importance of digital devices, we tried to provide one important step towards diagnosing common digital abilities. In the present paper, we introduced an assessment instrument for basic competencies in smartphone and tablet use, the DIGI. We demonstrated its feasibility and acceptability in healthy samples of different ages. Differences between older and younger adults were found particularly for navigation within apps and for producing motor-related errors. Only in older adults worse performance in handling traditional novel tools in the DILA-S went along with reduced digital tool competencies in the DIGI. We speculated that the overlap of digital tool competencies and novel tool use is due to shared correlates of potential rule retrieval.

Follow-up studies should evaluate the DIGI’s psychometric properties in larger groups including samples of healthy older participants as well as participants with cognitive impairments such as after suffering from a stroke. To further elucidate the underlying mechanisms of digital tool competencies, future studies should combine behavioral and neuroimaging techniques. When investigating digital tool competencies it appears particularly important to consider age and year of birth.

## Data availability statement

The raw data supporting the conclusions of this article will be made available by the authors, without undue reservation.

## Ethics statement

The studies involving humans were approved by University of Konstanz ethics committee. The studies were conducted in accordance with the local legislation and institutional requirements. The participants provided their written informed consent to participate in this study.

## Author contributions

SS: Conceptualization, Data curation, Formal analysis, Funding acquisition, Methodology, Project administration, Visualization, Writing – original draft. IB: Data curation, Methodology, Project administration, Writing – review & editing. KH: Data curation, Formal analysis, Visualization, Writing – original draft. JL: Data curation, Methodology, Writing – review & editing. VL: Writing – review & editing, Data curation. FG: Data curation, Writing – review & editing. CH: Data curation, Writing – review & editing, Conceptualization, Methodology. GK: Writing – review & editing. SK: Writing – review & editing. JR: Writing – review & editing, Funding acquisition, Methodology, Resources, Supervision.

## Footnotes

^1^https://www.moco.uni-konstanz.de/publikationen/assessments/
