# Supplementary material for: Diagnosing homo digitalis: towards a standardized assessment for digital tool competencies
Source: Front Psychol. 2024 Jan 4;14:1270437. doi: 10.3389/fpsyg.2023.1270437 (PMC10794727; doi:10.3389/fpsyg.2023.1270437)
Supplement: Supplementary file 1 [file Table_1.DOCX]

Post hoc power calculation

We computed statistical power (1-β) post hoc by use of the web page “http://powerandsamplesize.com/” (HyLown Consulting LLC, 2013–2023).

Power analyses were conducted for the smartphone and tablet selection, production, and error scores, thus resulting in six analyses. The results are displayed in the table below. The comparisons on the smartphone and tablet selection scores appeared to be underpowered. It needs to be noted that for the current sample, this variable came with low variance and at ceiling in both groups. The power in the remaining comparisons appeared to be acceptable.

| **Variable** | **Power (1-β)** | **N_B_** | **Sampling Ratio κ** | **α** | **MeanGroup ‘A’ (older)** | **MeanGroup ‘B’ (young)** | **Standard Deviation** |
| --- | --- | --- | --- | --- | --- | --- | --- |
| **Smartphone Selection** | 0.51 | 16 | 1 | 5% | 98.05 | 100.00 | 2.80 |
| **Smartphone Production** | 0.97 | 16 | 1 | 5% | 86.91 | 97.46 | 7.78 |
| **Smartphone Errors** | 0.87 | 16 | 1 | 5% | 2.50 | 0.06 | 2.23 |
| **Tablet Selection** | 0.22 | 16 | 1 | 5% | 98.21 | 99.55 | 3.20 |
| **Tablet Production** | 0.83 | 16 | 1 | 5% | 91.10 | 98.66 | 7.34 |
| **Tablet Errors** | 0.91 | 16 | 1 | 5% | 3.13 | 0.19 | 2.51 |
